# Reversal of Endothelial Dysfunction by GPBAR1 Agonism in Portal Hypertension Involves a AKT/FOXOA1 Dependent Regulation of H_2_S Generation and Endothelin-1

**DOI:** 10.1371/journal.pone.0141082

**Published:** 2015-11-05

**Authors:** Barbara Renga, Sabrina Cipriani, Adriana Carino, Michele Simonetti, Angela Zampella, Stefano Fiorucci

**Affiliations:** 1 Department of Surgical and Biomedical Sciences, University of Perugia, Perugia, Italy; 2 Department of Medicine, University of Perugia, Perugia, Italy; 3 Department of Pharmacy, University of Naples 'Federico II', Naples, Italy; University of Navarra School of Medicine and Center for Applied Medical Research (CIMA), SPAIN

## Abstract

**Background:**

GPBAR1 is a bile acids activated receptor expressed in entero-hepatic tissues. In the liver expression of GPBAR1 is restricted to sinusoidal and Kuppfer cells. In the systemic circulation vasodilation caused by GPBAR1 agonists is abrogated by inhibition of cystathione-γ-liase (CSE), an enzyme essential to the generation of hydrogen sulfide (H_2_S), a vasodilatory agent. Portal BAR501 is a semisynthetic bile acid derivative endowed with a potent and selective agonistic activity toward GPBAR1.

**Methods:**

Cirrhosis was induced in mice by carbon tetrachloride (CCL_4_) administration for 9 weeks. Liver endothelial dysfunction was induced by feeding wild type and Gpbar1^-/-^ mice with methionine for 4 weeks. In both models, mice were administered BAR501, 15 mg/kg/day.

**Results:**

By transactivation assay we demonstrate that BAR501 is a selective GPBAR1 agonist devoid of any FXR agonistic activity. In naïve rats, BAR501 effectively reduced hepatic perfusion pressure and counteracted the vasoconstriction activity of norepinephrine. In the CCl_4_ model, 9 weeks treatment with BAR501 effectively protected against development of endothelial dysfunction by increasing liver CSE expression and activity and by reducing endothelin (ET)-1 gene expression. In mice feed methionine, treatment with BAR501 attenuated endothelial dysfunction and caused a GPBAR1-dependent regulation of CSE. Using human liver sinusoidal cells, we found that modulation of CSE expression/activity is mediated by both genomic (recruitment of CREB to CRE in the CSE promoter) and non-genomic effects, involving a Akt-dependent phosporylation of CSE and endothelial nitric oxide (NO) synthase (eNOS). BAR501, phosphorylates FOXO1 and inhibits ET-1 transcription in liver sinusoidal cells.

**Conclusions:**

BAR501, a UDCA-like GPBAR1 agonist, rescues from endothelial dysfunction in rodent models of portal hypertension by exerting genomic and non-genomic effects on CSE, eNOS and ET-1 in liver sinusoidal cells.

## Introduction

Bile acids are amphipatic molecules synthesized in the liver from oxidation of cholesterol. Beside their role in nutrient absorption, primary bile acids, chenodeoxycholic acid (CDCA) and cholic acid (CA), and secondary bile acids, deoxycholic acid (DCA) and lithocholic acid (LCA), and their glycine and taurine conjugates, act as signaling molecules by activating a family of receptors collectively known as the “bile acid activated receptors” (BARs) [1–3]. The G protein coupled receptor, GPBAR1 (also known as TGR5) is a cell surface receptor highly expressed by non parenchimal liver cells [3], enterocytes and endocrine intestinal cells [1,2]. GPBAR1 mediates non-genomic activities of secondary bile acids by increasing intracellular concentrations of cAMP, leading to downstream activation of cAMP-response element (CRE)-binding proteins (CREBs) in target cells.

Portal hypertension is a common complication of liver cirrhosis. Importantly, while liver fibrosis represents the main causative factor involved in development of increased intrahepatic resistance, a vasculogenic component contributed to by an insufficient production of NO by LSEC, endothelial dysfunction, makes also an important contribution, [4–6]. The endothelial dysfunction occurring in liver cirrhosis, leads to enhanced contraction of activated perisinusoidal hepatic stellate cells (HSC) and increased intrahepatic vascular resistance to portal flow [4–6].

Along with resident macrophages (Kuppfer cells), the liver sinusoidal endothelial cells (LSEC) express GPBAR1, whose activation regulates the activity of endothelial nitric oxide (NO) synthase (eNOS) [3], suggesting a potential role for this receptor in the treatment of endothelial dysfunction in the setting of liver cirrhosis. One conundrum in targeting eNOS in LSEC, however, is the fact that impaired NO generation in liver cirrhosis is largely due to an impaired Akt signaling along with excessive sequestration of the eNOS protein by caveolin 1. Both mechanisms contribute to a reduced eNOS activity, while the expression of the gene and the protein is generally conserved [7–9].

We have recently shown, that in arterial (HAEC) end venular (HUVEC) endothelial cells, GPBAR1 agonism increases the expression/ activity of cystathione-γ-liase (CSE, CTH, EC 4.4.1), a key enzyme in the “trans-sulfuration pathway” that generates hydrogen sulfide (H_2_S), a vasodilatory agent [10]. Regulation of CSE by GPBAR1 ligands occurs trough genomic and genomic mechanisms. Molecular analysis has revealed that the CSE promoter contains two functional CRE and that CREB is recruited to these CRE upon activation of GPBAR1 by LCA [10].

Alterations of the trans-sulfuration pathway are relatively common in liver cirrhosis, with two/third of cirrhotic patients developing a hyper-homocysteinemia regardless the etiology of liver damage [11–14]. In the systemic circulation homocysteine promotes endothelial dysfunction by impairing eNOS activity in endothelial cells [15]. Alterations of the trans-sulfuration pathway induced by a methionine feeding in rodents leads to hyper-homocysteinemia, reduced generation of H_2_S and increased intrahepatic resistance to portal flow [15–17]. Previous studies have shown that in the liver CSE expression/activity is increased by Farnesoid-x-receptor (FXR) ligands [18] but, whether GPBAR1 protects against development of endothelial dysfunction in rodent models of portal hypertension is unknown.

**Fig 1:**
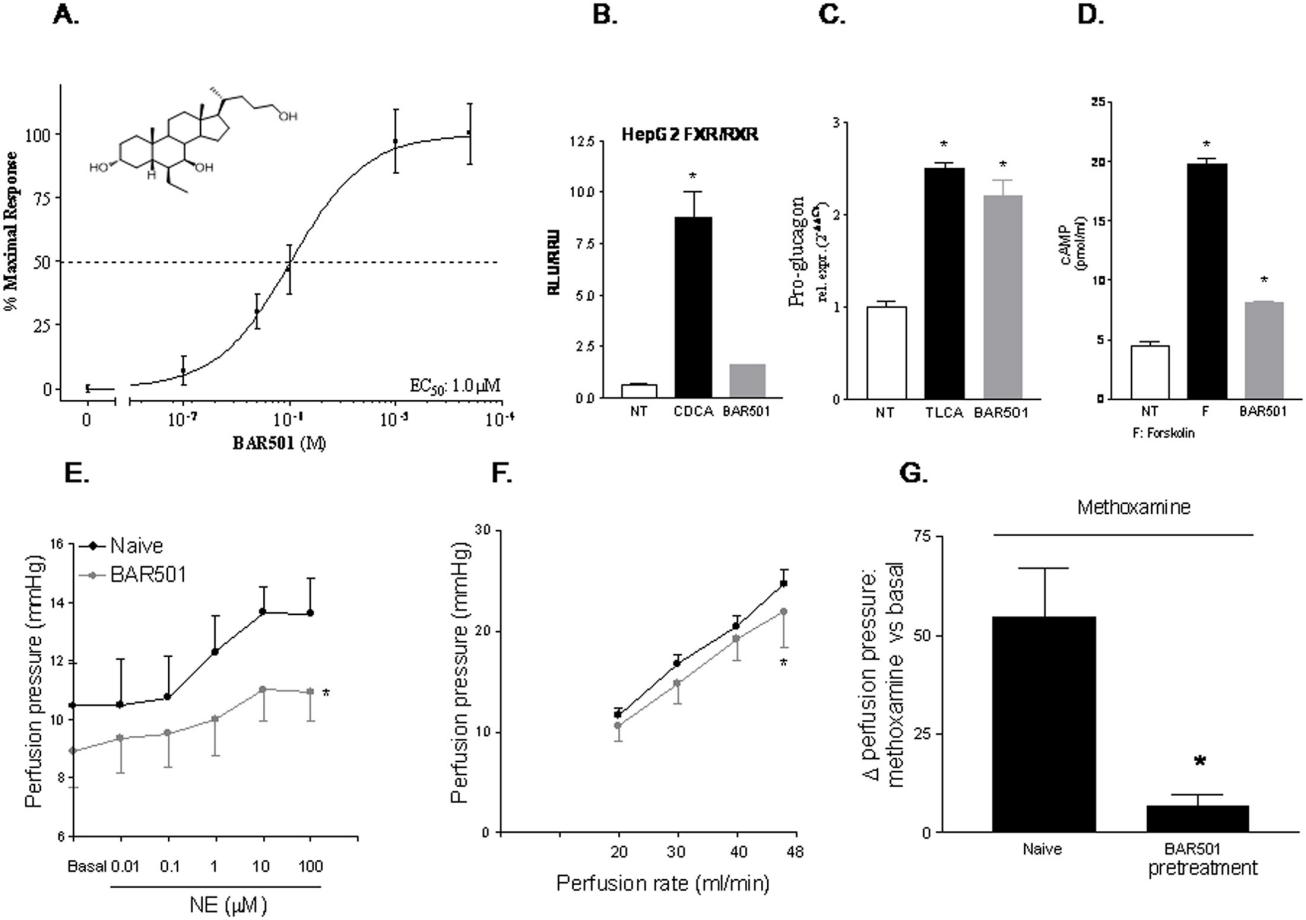
Pharmacological characterization of BAR501. (A) Effect of BAR501 on GPBAR1 transactivation. BAR501 causes a concentration-dependent transactivation of GPBAR1 in HEK293 T cells transfected with a CREB responsive element. Data are mean ± SE of 3 experiments. (B) BAR501 fails to promote FXR transactivation. (C) Effect of BAR501 on mRNA expression of pro-glucagon in GLUTAg cells. Data are mean ± SE of 3 experiments. *p<0.05 versus not treated cells. (D) Effect of BAR501 on cAMP production in GLUTAg cells. Results are the mean ± SE of 3 experiments. *p<0.05 versus not treated cells (NT). F is forskolin. (E) Effect of BAR501 on vasomotor activity of nor-epinephrine (NE) on rat liver. Administering rats with 15 mg/kg/day BAR501 for 6 days attenuated the vasomotor response to NE at any concentration tested. (F) Effect of BAR501 on liver perfusion pressure caused by shear stress in rats. Data are mean ± SE of 4–6 animals per group. *p<0.05 versus shear stress alone (G) BAR501 attenuates vasomotor response to methoxamine in rats. Data are mean ± SE of 7 livers. *p < 0.05 versus methoxamine alone.

The 6β-ethyl-3a,7b-dihydroxy-5b-cholan-24-ol (BAR501) is a UDCA derivative that selectively activates GPBAR1 (Fig 1A). In the present study, by using pharmacological and genetic approaches we demonstrate that GPBAR1 activation protects against development of endothelial dysfunction in rodent models of portal hypertension, providing a molecular ground for the exploitation of GPBAR1 ligands in the treatment of portal hypertension in liver disorders.

## Materials and Methods

### Chemicals

BAR501 was synthesized as described elsewhere [19]. Norepinephrine (NE), L-methionine, methoxamine, TLCA, oleanolic acid, betulinic acid, UDCA and LY294002 were from Sigma Aldrich (Milan, Italy).

### Animals

C57BL6 and male Wistar rats were from Harlan Nossan (udine, Italy). GPBAR1 null mice (generated directly into C57BL/6NCrl background), and congenic littermates on C57BL/6NCrl mice were originally donated by Dr. Galya Vassileva (Schering-Plough Research Institute, Kenilworth) [20]. The colonies were maintained in the animal facility of University of Perugia. Mice were housed under controlled temperatures (22°C) and photoperiods (12:12-hour light/dark cycle), allowed unrestricted access to standard mouse chow and tap water and allowed to acclimate to these conditions for at least 5 days before inclusion in an experiment. A total number of 96 mice and 21 rats were used in this study. The study was conducted in agreement with the Italian law and the protocol was approved by a ethical committee of University of Perugia and by a National committee of Ministry of Health (permission n. 245/2013-B). The health and body conditions of the animals were monitored daily by the veterinarian in the animal facility. The study protocol caused minor suffering, however, animals that lost more than 25% of the initial body weight were euthanized. At the day of sacrifice (prior to measure the portal pressure the animals (mice) were deeply anesthetised with a mixture of tiletamine hypochoride and zolazepam hypocloride/xylazine at a dose of 50/5 mg/Kg. Similarly rats, at the day of sacrifice rats were deeply anesthetised with a lethal dose tiletamine hypochoride and zolazepam hypocloride/xylazine and liver were isolated and perfused.

### Animal models

Liver cirrhosis was induced by carbon tetrachloride (CCl_4_) administration. For this purpose, C57BL6 mice (40 animals) were administered i.p. 500 μL/Kg body weight of CCl_4_ in an equal volume of paraffin oil twice a week for 9 weeks. CCL_4_ mice were randomized to receive BAR501 (15 mg/Kg daily by gavage) or vehicle (distilled water). In another experimental setting, wild type C57BL6 mice were administered 500 μl/Kg body weight of CCl_4_ in an equal volume of paraffin oil twice a week for 3 weeks. CCL_4_ mice were then randomized to receive BAR501 (30 and 45 mg/Kg daily by gavage) or vehicle (distilled water). Serum bilirubin, albumin, aspartate aminotransferase (AST), alanine aminotransferase (ALT) and alkaline phosphatase (ALP) were measured by routine biochemical clinical chemistry (S2 Table). For histological examination, portions of the right and left liver lobes were fixed in 10% formalin, embedded in paraffin, sectioned and stained with Sirius red and Hematoxylin/Eosin (H&E).

In a further model, moderate hyperhomocysteinemia [15] was induced in Gpbar1^+/+^ and Gpbar1^-/-^ mice (56 animals) by administration of L-methionine (1 g/kg daily by gavage) for a period of 4 weeks (n = 20). Mice administered L-methionine were randomized to receive BAR501 (20 mg/Kg daily by gavage) or vehicle (distilled water).

### Isolated and perfused rat liver preparation

To investigate the effect of BAR501 on intrahepatic microcirculation, naïve rats were administered BAR501 (15 mg/Kg daily by gavage) or water for 6 days. At the end of this period, analysis of hepatic vascular responses to NE (from 0.01 to 100 μmol/L) or methoxamine (100 μM) was performed using the isolated and perfused rat liver preparation, as described previously [15–18]. The vasomotor responses to changes of liver flow (shear stress) was measured as described previously [15–18,21]. During these studies, the global viability of livers was assessed by standard criteria: i.e. inspection of gross appearance, stable pH of the perfusate, stable perfusion pressure for 20 min and bile flow >1 μl/min per g liver. The flow rate during each individual perfusion was maintained at a constant rate of 20 ml/min (S1 Table).

### Cell cultures

Human liver sinusoidal cells (LSEC) were from Innoprot (cat. N° P10652; Barcelona, Spain). LSEC were cultured in endothelial cell medium (Innoprot) additioned with 5% fetal bovine serum, endothelial cell growth supplement (ECGS) (Innoprot) and antibiotics. HepG2 (HB-8065), THP1 (TIB-202) and HEK293T (CRL-1573) cell lines were from ATCC (Manassas, VA; USA). HepG2 cells were cultured in E-MEM supplemented with 10% FBS, 1% glutamine, 1% penicillin/streptomycin. HEK293T cells were cultured in DMEM supplemented with 10% FBS, 1% glutamine, and 1% penicillin/streptomycin. GLUTAg cells, a murine intestinal endocrine cell line, were kindly donated by Dr. D. J. Drucker, Banting and Best Diabetes Centre, University of Toronto, Toronto, Canada, and cultured in D-MEM, supplemented with 10% FBS, 1% glutamine, and 1% penicillin/streptomycin.

### Transactivation assay

For FXR mediated transactivation, HepG2 cells were plated at 5 × 10^4^ cells/well in a 24 well plate. Cells were transfected with 200 ng of the reporter vector p(hsp27)-TK-LUC containing a FXR response element (IR1) cloned from the promoter of heat shock protein 27 (hsp27), 100 ng of pSG5-FXR, 100 ng of pSG5-RXR, and 100 of pGL4.70 (Promega), a vector encoding the human Renilla gene. For GPBAR1 mediated transactivation, HEK-293T cells were plated at 1 × 10^4^ cells/well in a 24 well-plate and transfected with 200 ng of pGL4.29 (Promega), a reporter vector containing a cAMP response element (CRE) that drives the transcription of the luciferase reporter gene luc2P, with 100 ng of pCMVSPORT6-human GPBAR1, and with 100 ng of pGL4.70. At 24 h post-transfection, HepG2 and HEK293T cells were incubated with 10 μM BAR501 for 18 h and luciferase activities were assayed and normalized against the Renilla activities. For CRE1 mediated transactivation HEK-293T cells were transfected with 200 ng of pGL4(CRE1)_5X_, 100 ng of pCMVSPORT6-human GPBAR1 and 100 ng of pGL4.70 (a vector encoding the human Renilla gene). Forty-eight hr post-transfection, cells were stimulated 18 hr with a dose response of BAR501 (1, 10, 25 and 50 μM) (S1 Table).

### cAMP assay

cAMP concentrations were assayed using the Direct Cyclic AMP enzyme immuno-assay kit (Arbor Assay cat. no. K019-H1). For this assay, GLUTAg cells were serum starved overnight and then stimulated for 30 min with 10 μM forskolin (F) or BAR501 (S1 Table).

### RNA extraction and Real-Time PCR

Total RNA was isolated from LSEC or tissues using the TRIzol reagent according to the manufacturer’s specifications (Life Technologies). One microgram of RNA was purified from genomic DNA by DNase-I treatment (Life Technologies) and reverse-transcribed using random hexamer primers with Superscript-II (Life Technologies) in a 20-μL reaction volume. Ten ng cDNA were amplified in a 20 μl solution containing 200 nM of each primer and 10 μl of KAPA SYBR FAST Universal qPCR Kit (KAPA BIOSYSTEMS). All reactions were performed in triplicate, and the thermal cycling conditions were as follows: 3 min at 95°C, followed by 40 cycles of 95°C for 15 s, 56°C for 20 s and 72°C for 30 s. The relative mRNA expression was calculated accordingly to the Ct method. PCR primers were designed using the software PRIMER3 (http://frodo.wi.mit.edu/primer3/) using published data obtained from the NCBI database. Forward and reverse primer sequences were as follows: hGAPDH: gaaggtgaaggtcggagt and catgggtggaatcatattggaa; hCSE: cactgtccaccacgttcaag and gtggctgctaaacctgaagc; hCBS: tcgtgatgccagagaagatg and ttggggatttcgttcttcag; hTGR5: cactgttgtccctcctctcc and acactgctttggctgcttg; heNOS: agtgaaggcgacaatcctgtat and agggacaccacgtcatactcat; hET1: agggctgaagacattatggaga and cctggtttgtcttaggtgttcc; mGAPDH: ctgagtatgtcgtggagtctac and gttggtggtgcaggatgcattg; mpro-glucagon: tgaagacaaacgccactcac and caatgttgttccggttcctc; mTGFb1: ttgcttcagctccacagaga and tggttgtagagggcaaggac; mCOL1A1: acgtcctggtgaagttggtc and cagggaagcctctttctcct; maSMA: tgtgctggactctggagatg and gaaggaatagccacgctcag; mTNFa:acggcatggatctcaaagac and gtgggtgaggagcacgtagt; mTGR5: ggcctggaactctgttatcg and gtccctcttggctcttcctc; mIL1b: tcacagcagcacatcaacaa and tgtcctcatcctcgaaggtc; mCBS: agaagtgccctggctgtaaa and caggactgtcgggatgaagt; mCSE: tgctgccaccattacgatta and gatgccaccctcctgaagta; meNOS: agaagagtccagcgaacagc and tgggtgctgaactgacagag; miNOS: acgagacggataggcagaga and cacatgcaaggaagggaact; mET1: tgccaagcaggaaaagaact and acgaaaagatgccttgatgc; mCAV1: ttgaagatgtgattgcagaacc and tcgtagacaacaagcggtaaaa (S1, S3, S4–S6 Tables).

### CSE activity

CSE activity was measured in liver tissues or in serum starved LSEC administered with 10 μM TLCA or BAR501 for 24 and 48 hr according to a previously published method [18] (S3 Table).

### Nitrite/Nitrate

Hepatic nitrate/nitrite concentrations were measured by a colorimetric assay (Cayman Chemical, Ann Arbor, Michigan; USA) (S3 Table).

### Chromatin Immunoprecipitation (ChIP)

Detailed methods for ChIP protocol and Real-Time data analysis have been described previously [21]. Briefly, 10 x 10^6^ serum starved SEC cells were stimulated 18 hr with 10 μM BAR501 or received the vehicle alone (1% DMSO). Chromatin was immunoprecipitated with an anti-phospho-CREB antibody (Santa Cruz) or with an anti-IgG as negative control. The sequences of primers used for the amplification of the human CREB responsive sequence CRE1s were: ctggtctcgaactcttgacttcag and gctaacgcctattaatcccagcac (S5 Table).

In another experimental setting LSEC cells were exposed to 10 μM BAR501 for 18 hr. Chromatin was immunoprecipitated with an anti-phospho-FOXO1 antibody (Santa Cruz) or with an un-relevant anti-IgG as negative control. The sequences of primers used for the amplification of the human FOXO consensus sequence were: gcctgttggtgactaataacac and cagctctgccggctttttatat (S6 Table).

### Immunoprecipitation

Overnight serum starved LSEC cells were exposed to BAR501 (10 μM) for 0, 5, 15, 30 and 60 min. After stimulation, cells were washed with cold PBS and lysed in 500 μl E1A lysis buffer containing protease and phosphatase inhibitors. Lysates were sonicated and clarified by centrifugation at 13,000*g* for 10 min, and the protein concentrations was measured by Bradford assay. Five-hundred μg total proteins were pre-cleared on a rotating wheel for 1 h at 4°C using protein A Sepharose beads (Amersham Biosciences) and 1 μg of irrelevant antibody of the same species and isotype as CSE. Immunoprecipitation was performed overnight at 4°C with 1 μg CSE antibody (Santa Cruz) or anti-IgG as a negative control antibody in the presence of 40 μl of protein A Sepharose (Amersham Biosciences). The resultant immunoprecipitates were washed five times with 1 ml of E1A lysis buffer and then used for Western blotting.

### Western blotting

To investigate protein expression of GPBAR1 and CSE, LSEC were serum starved overnight and then stimulated with 10 μM BAR501 for 18 h. In another experimental setting serum starved LSEC were incubated 5, 15, 30 and 60 minutes with 10 μM BAR501. Total lysates were prepared by solubilization of endothelial cells in E1A lysis buffer containing protease and phosphatase inhibitors. The proteins were separated by SDS-PAGE, transferred to nitrocellulose membranes (Bio-Rad) and probed with primary antibodies CSE (Santa Cruz), GPBAR1/TGR5 (Abcam), tubulin (Sigma), phospho-Akt (Thr308—Santa Cruz), Akt (Santa Cruz), phospho-Serine (Abcam), phosphoeNOS (ser1177 –Cell Signaling), eNOS (Cell Signaling), phosphoFOXO1 (Thr24 –Santa Cruz) and FOXO1 (Santa Cruz). Nitrocellulose membranes from immunoprecipitation (IP) experiments were first probed with a phospho-serine antibody, stripped and then re-probed with the CSE antibody. Similarly, nitrocellulose membranes from IP experiments were first probed with the phospho-Akt antibody, stripped and then re-probed with the Akt antibody. The anti-immunoglobulin G horseradish peroxidase conjugate (Bio-Rad) was used as the secondary antibody, and specific protein bands were visualized using Super Signal West Dura (Pierce), following the manufacturer’s suggested protocol.

### Statistical analysis

All values are mean ± Standard Error (SE) of *number (n)* observations per group. Comparisons of more than two groups were made by one-way ANOVA with post-hoc Tukey’s test. The Student’s t-test for unpaired data was used when appropriate.

## Results

### BAR501 is a selective GPBAR1 agonist and reduces hepatic vasoconstriction caused by NE

BAR501 (structure in Fig 1A) is a selective GPBAR1 ligand [19,22]. Indeed, as shown in Fig 1A and 1B, while it failed to transactivate FXR in HepG2 cells overexpressing a FXRE, the compound effectively transactivates GPBAR1 in HEK293 cells overexpressing a CRE along with GPBAR1, with a EC_50_ of 1 μM (Fig 1A and 1B, *p<0.05 versus not treated cells).

To further confirm that BAR501 activates GPBAR1 directly, we have assessed whether it boosted pro-glugagon-1 gene (GLP-1) expression by GLUTAg cells, a murine endocrine cell line that express high levels of GPBAR1 [23]. As shown in Fig 1C and 1D, exposure of GLUTAg to BAR501 (10 μM) increased the expression of GLP-1 mRNA by 2.5 folds. This effect associated with a robust increase of cAMP concentrations (Fig 1C and 1D, p<0.05 versus control cells). Taken together these data indicate that BAR501 is a selective GPBAR1 agonist devoid of FXR agonistic activity.

Because in the liver the expression of GPBAR1 is restricted to LSEC, we have then examined whether BAR501 effectively modulates LSEC function *in vivo* by assessing intrahepatic resistance to portal flow using the model of rat liver isolated and perfused [15, 16, 20]. Concentration–response curves to NE were investigated at a constant perfusion flow rate of 20 mL/min. As shown in Fig 1E, addition of NE to the perfusion solution resulted in a concentration-dependent increase in portal perfusion pressure, that reached the maximum at 10 μM. Pretreating rats for 6 days with BAR501, 15 mg/kg, reduced basal portal pressure and blunted the vasoconstriction activity of NE (Fig 1E, *p<0.05 versus baseline, *P>0.05 versus NE alone). Additionally, pretreatment with BAR501 attenuated the hepatic vasomotor activity induced by shear stress (Fig 1F, *P<0.01) and methoxamine (Fig 1G, *p<0.05).

### BAR501 protects against development of portal hypertension in the CCL_4_ model

**Fig 2:**
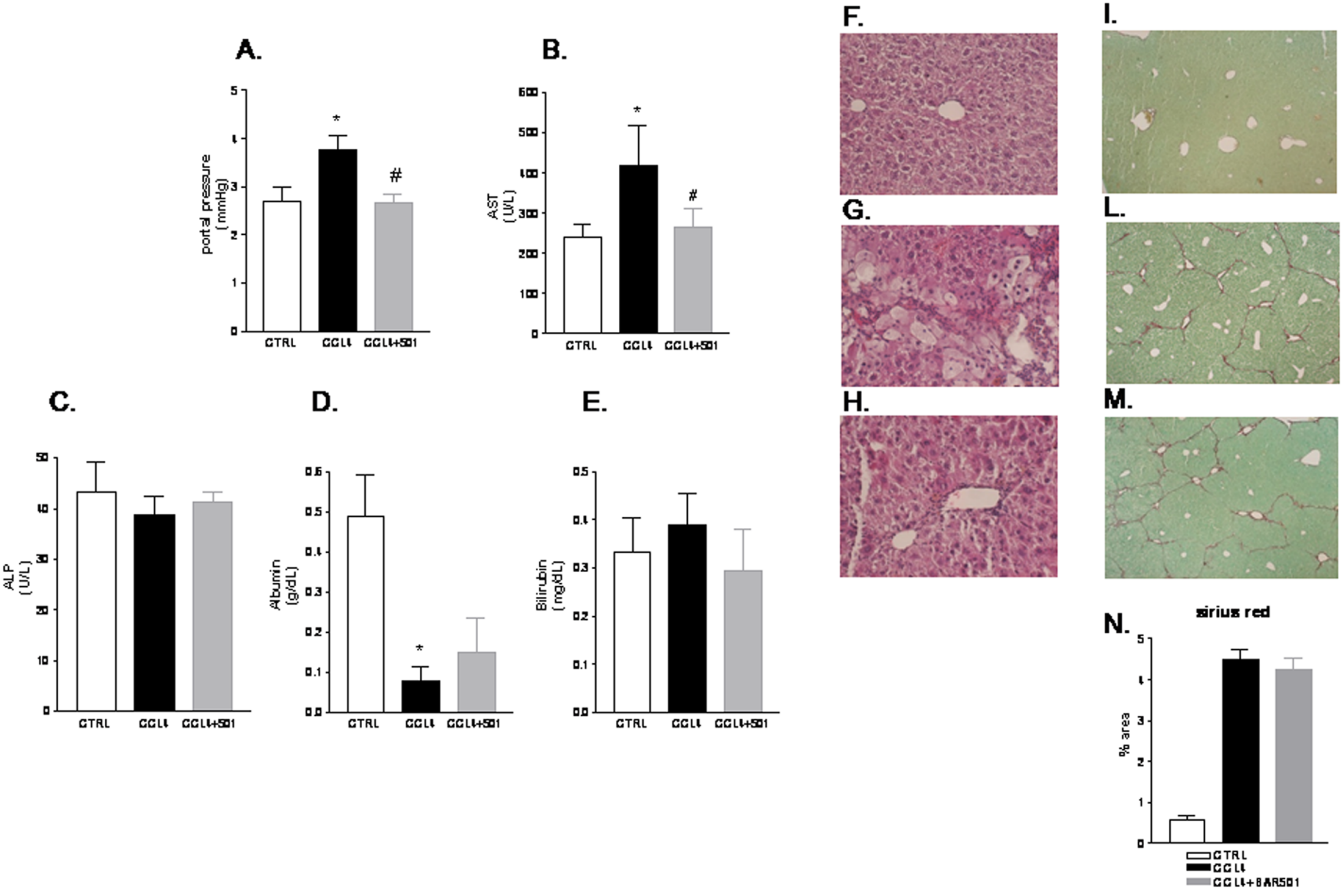
Administration of BAR501 exerts a direct vasodilatory activity in the CCl_4_ model. Effect of BAR501 on (A) portal pressure, (B) AST, (C) alkaline phosphatase (ALP), (D) Albumin, (E) Bilirubin in mice renedered cirrhotic by administration with CCl_4_. (F-H) Hematoxylin and eosin (H&E) staining. (I-M) Syrius red staining. (N) Image J quantification of Syrius red staining. Data are mean ± SE of 8–10 animals per group. *p<0.05 versus naïve mice. #p<0.05 versus CCl_4_.

We have next investigated whether BAR501 modulates the hepatic microcirculation in mice administered CCl_4,_ a model of liver cirrhosis/fibrosis. As shown in Fig 2, mice administered CCl_4_ for 9 week developed a severe liver injury (AST levels), liver fibrosis (Sirius red staining) and impaired biosynthetic function (reduced albumin levels) (*p<0.05 versus naïve mice). Additionally, CCL_4_ administration increased intrahepatic resistance, as measured by direct cannulation of portal vein (Fig 2A; p<0.05 versus naive mice)

Treating mice with BAR501 at the dose of 15 mg/Kg reduced portal pressure and AST plasma levels, thought it had no effect on serum biochemistry including alkaline phosphatase, albumin and bilirubin (Fig 2A–2E). Morphometric analysis performed on liver section stained with H&E and Sirius red revealed that while a bridging fibrosis developed in CCl_4_-treated mice (Fig 2, panels F-H and I-N), these changes were only minimally affected by BAR501.

**Fig 3:**
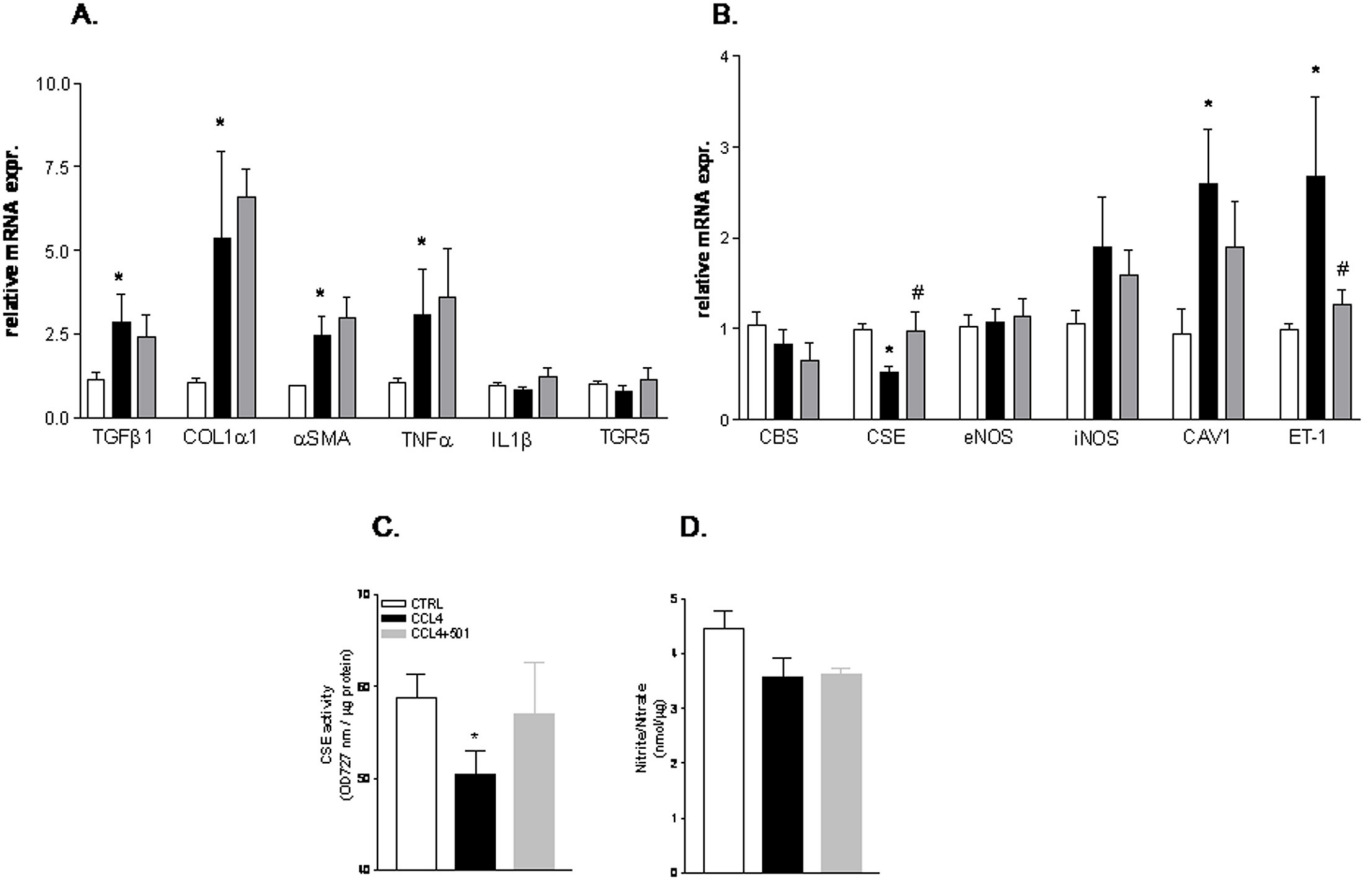
BAR501 regulates the expression of CSE and ET-1 in the CCl_4_ model of liver cirrhosis. C57BL6 mice were treated for 9 weeks with CCl_4_ or with the combination of CCl_4_ plus BAR501 15 mg/kg. The relative hepatic mRNA expression of TGFβ1, COL1α1, αSMA, TNFα, IL1β, GP-BAR1 (panel A) and CBS, CSE, eNOS, iNOS, CAV-1 ET-1 (panel B) was assayed by Real-Time PCR. (C) Effect of BAR501 on CSE activity in the CCl_4_ model. (D) Effect of BAR501 on nitrite/nitrate in CCl_4_ treated mice. Results are the mean ± SE of 4–8 mice per group. *p<0.05 versus naive mice. #p<0.05 versus CCl_4_ alone.

Quantitative Real-Time PCR analysis of liver homogenates confirmed these biochemical and morphometric observations. Thus, as illustrated in Fig 3A, administration of mice rendered cirrhotic by CCl_4_ with BAR501 failed to reverse the expression of pro-fibrogenic (i.e. TGFβ1, Col1α1 and α-SMA) and pro-inflammatory markers (i.e. TNFα). Similar findings were observed in mice administered CCl_4_ in combination with BAR501 at doses of 30 and 45 mg/Kg (S1A–S1F Fig).

### BAR501 attenuates endothelial dysfunction by regulating CSE expression/activity

Because the above mentioned data indicate that BAR501 exerts portal pressure-lowering effects but has no anti-fibrogenetic activities, suggesting a potential role of this agent in regulating endothelial mechanisms in the liver microcirculation, we have investigated markers of endothelial dysfunction in mice administered with CCl_4_ alone or in combination with BAR501. As shown in Fig 3B, RT-PCR analysis demonstrated that only the relative mRNA expression of CSE, but not that of CBS, eNOS and iNOS, was significantly decreased in mice exposed to CCl_4_ compared to control animals and that administration of BAR501 reversed this pattern (Fig 3B, *p<0.05 versus control mice, #p<0.05 versus CCl_4_). The quantification of CSE activity and nitrites/nitrates levels in liver specimens obtained from mice administered BAR501 confirmed RT-PCR data, being liver activity of CSE markedly reduced in CCL_4_ treated mice (Fig 3C, *p<0.05 versus control mice). This pattern was reversed by treating animals with BAR501. Interestingly, the GPBAR1 ligand increased CSE expression and activity but had no effect on nitrite/nitrate concentrations (Fig 3C and 3D, *p<0.05 versus control mice). Because, impaired eNOS activity in the CCL_4_ model has been ascribed to enhanced binding by caveolin-1, we have examined the effect of the GPBAR1 ligand on this gene. As shown in Fig 3B, caveolin-1 gene expression increased dramatically in CCL_4_ treated mice and this pattern was only slightly affected by exposing CCL_4_ mice to BAR501.

Endothelin-1 (ET-1) is a potent vasoconstrictor produced in the liver by LSEC. Noteworthy, while ET-1 gene expression was markedly upregulated in mice administered CCL_4_ alone, the regulation of this gene was potently reversed by BAR501.

### BAR501 corrects endothelial dysfunction caused by L-methionine feeding in mice

**Fig 4:**
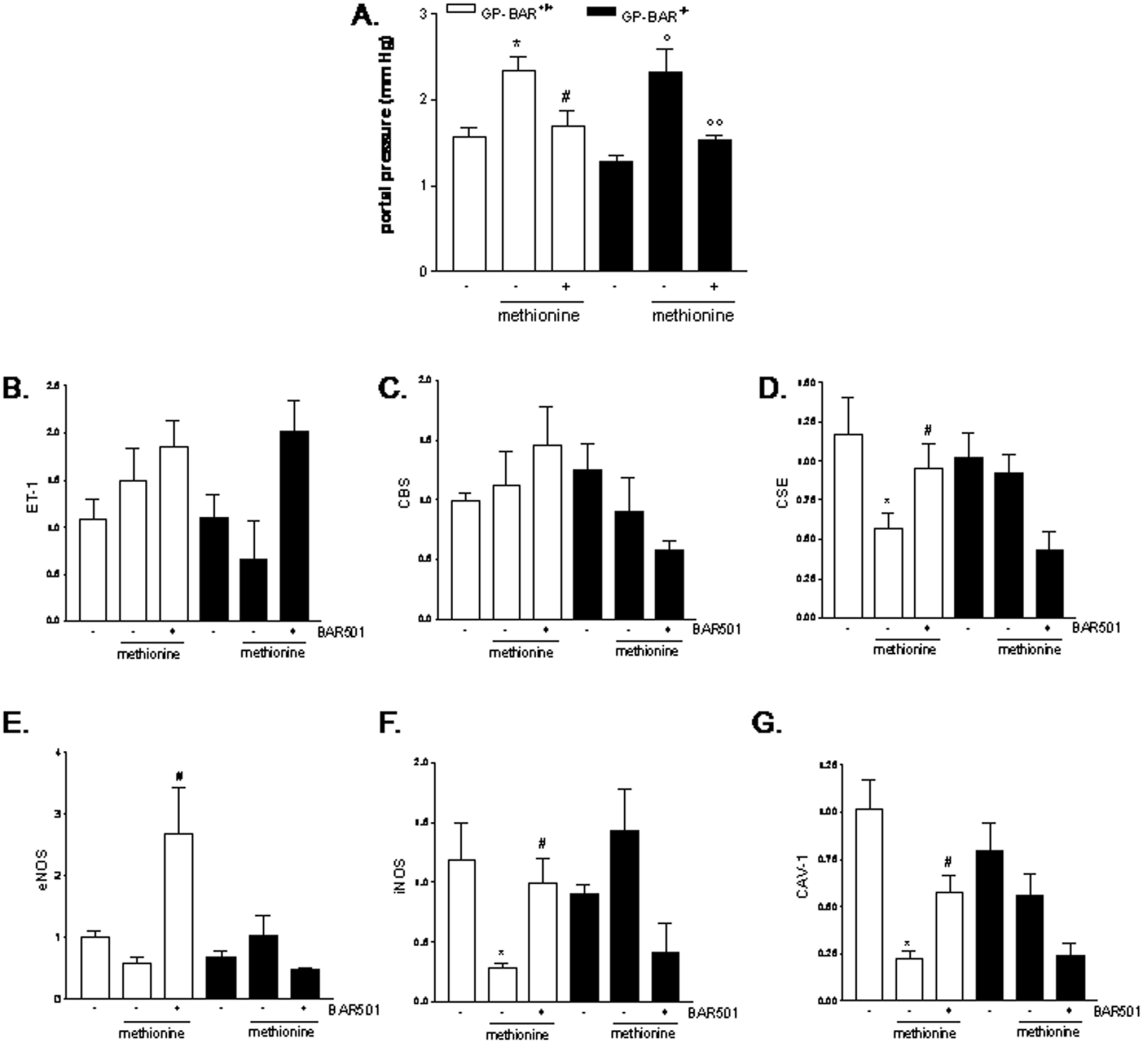
BAR501 correct for endtothelial dysfunction caused by methionine diet. Gpbar1^+/+^ and Gpbar1^-/-^ mice administered L-methionine for 4 weeks were randomized to receive BAR501 (20 mg/Kg daily by gavage) or vehicle (distilled water). Effect of BAR501 on (A) portal pressure, (B) ET-1 mRNA, (C) CBS mRNA, (D) CSE mRNA, (E) eNOS mRNA, (F) iNOS mRNA and (G) CAV-1. Results are the mean ± SE of 4–8 mice per group. *p<0.05 versus pbar1^+/+^ mice. #p<0.05 versus Gpbar1 ^+/+^ mice fed methionine. °p<0.05 versus Gpbar1^-/-^ mice. °°p<0.05 versus Gpbar1^-/-^ mice fed methionine.

Because these data highlight that activation of GPBAR1 *in vivo* resets the molecular mechanisms involved in development of endothelial dysfunction in the CCL_4_ model, we have then examined whether BAR501 has the ability to counteract endothelial injury in Gpbar1^+/+^ and Gpbar1^-/-^ animals administered with methionine for 4 weeks. The methionine feeding induces a severe endothelial dysfunction leading to increased intra-hepatic resistance to portal flow [15]. Of interest, administration of BAR501 significantly reduced resistance to portal flow caused by methionine intake in both Gpbar1^+/+^ and Gpbar1^-/-^ animals (Fig 4A, #p<0.05 versus Gpbar1^+/+^ mice fed methionine; °°p<0.05 versus Gpbar1^-/-^ mice fed methionine).

Administering Gpbar1^+/+^ mice with methionine diet had no effect on the expression of ET-1, CBS, eNOS and CAV-1, while the expression of CSE and iNOS genes was significantly down-regulated (Fig 4C–4H, *p<0.05 versus GPBAR^+/+^ mice). Treating Gpbar1^+/+^ with BAR501 increased the expression of CSE, eNOS and iNOS (Fig 4C–4H, #p<0.05 versus Gpbar1^+/+^ mice fed methionine). However, BAR501 failed to regulate CSE, eNOS and iNOS in Gpbar1^-/-^ mice, indicating that these genes are directly regulated by GPBAR1 *in vivo* (Fig 4C–4H).

### BAR501 regulates CSE expression/activity activity in human LSEC

**Fig 5:**
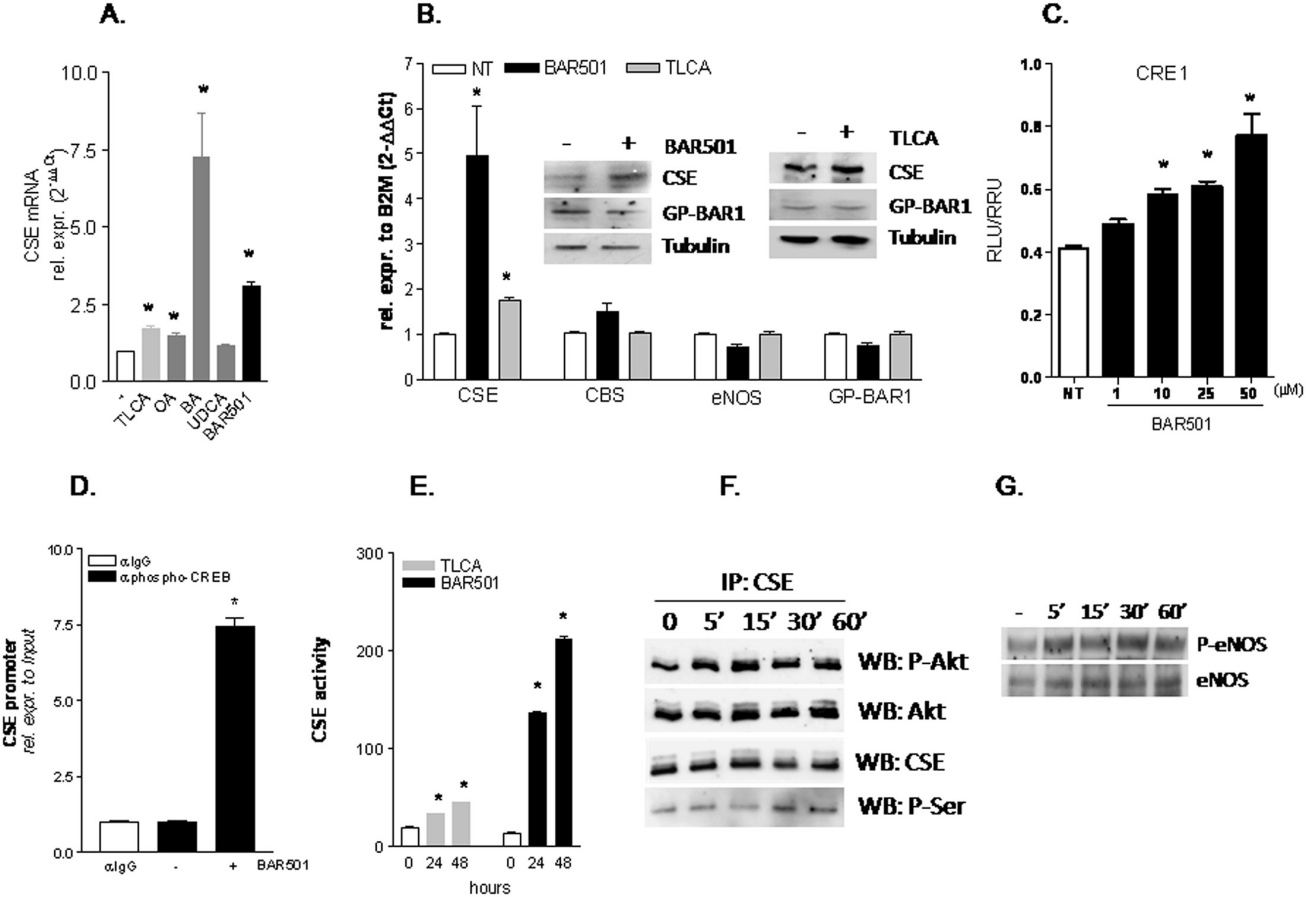
GPBAR1 activation by BAR501 modulates the expression/activity of regulates CSE in human LSEC. (A) Serum starved LSEC were exposed to 10 μM TLCA, OA, BA, UDCA or BAR501 for 18 hr. CSE mRNA expression was evaluated using Real-Time PCR. (B) Serum starved LSEC were exposed to 10 μM TLCA or BAR501 for 18 hr. Relative mRNA expression of CSE, CBS, eNOS and TGR5 was assayed by Real-Time PCR. Protein expression of CSE, GPBAR1 and tubulin was measured by Western blotting. (C) LSEC were transiently transfected with pCMVSPORT-hTGR5 and pGL4(CRE1)_5X_ as described in *Materials and Methods*. Forty-height h post-transfection cells were stimulated 18 h with BAR501 (10 μM). (D) ChIP assay carried out in LSEC left untreated or primed with BAR501 as described in *Materials and Methods*. RT-PCR was performed with specific primers flanking the responsive element CRE1 on human CSE promoter. (E) CSE activity in LSEC administered 10 μM TLCA or BAR501 for 24 and 48 h. (F) Effect of BAR501 on phosphorylation of CSE on serine residues. Serine phosphorylation of CSE was assessed by immunoprecipitation of CSE followed by Western blot determination of phosphoserine and phospho-Akt1 content in SEC exposed to BAR501 (10 μM) for 0, 5, 15, 30, and 60 min. (G) Exposure of LSEC to BAR501 increases eNOS phosphorylation. All analyses were carried out in triplicate and the experiments were repeated twice. *p<0.05 versus not treated cells.

Serum starved LSEC were exposed to GPBAR1 ligands including TLCA, oleanolic acid, betulinic acid, UDCA and BAR501. As shown in Fig 5A, RT-PCR analysis of CSE mRNA revealed that all these agents, but UDCA, increased the expression of CSE mRNA, with betulinic acid being by far the most potent (Fig 5A, *p<0.05 versus not treated cells). Importantly, while incubating LSEC with TLCA or BAR501 (10 μM) increased the expression of CSE, mRNA and protein, the two agents had no effect on the relative expression of CBS, eNOS and GPBAR1 (Fig 5B, *p<0.05 vs NT cells).

We have recently shown that the CSE promoter contains two conserved CRE (CRE1 and CRE2) and demonstrated that both these CRE were responsive to GPBAR1 activation in HUVEC [10]. Consistent with these observations, results from luciferase reporter gene assay conducted in HEK293T cells transiently transfected with GPBAR1 and a pGL4 vector containing five copies of CRE1 demonstrated that the activation of this CRE by BAR501 was concentration-dependent (Fig 5C, *p<0.05 versus NT cells). To confirm that the induction of CSE gene expression in LSEC was CREB dependent, we performed a ChIP experiment. The results of these studies demonstrated that the interaction of CREB to the CSE promoter occurs in basal conditions and is strongly increased following treatment with BAR501 (Fig 5D, *p<0.05).

The exposure of LSEC to BAR501 also regulates CSE activity (Fig 5E, *p<0.05). Because we have previously shown, that CSE activity might be regulated by Akt through phosphorylation on Ser^377^ (10), we have next investigated whether BAR501 changes the CSE phosphorylation status in these cells. Results from IP experiments demonstrated the existence of a protein complex between the CSE and Akt1 which is assembled already in basal conditions (Fig 5F). Of interest, the interaction between CSE and Akt1 (both phosphorylated or total) in LSEC increased after 5 min of incubation with BAR501 (Fig 5F). Moreover, 30 min treatment with BAR501 increased the phosphorylation of CSE on serine residues without affecting total CSE protein levels (Fig 5F).

Because eNOS phosphorylation is Akt dependent, we have then investigated whether GPBAR1 activation in LSEC regulates eNOS phosphorylation, and, as shown in Fig 5G, found that exposure to BAR501, caused a time-dependent phosphorylation of eNOS.

### BAR501 downregulates ET-1 in LSEC

**Fig 6:**
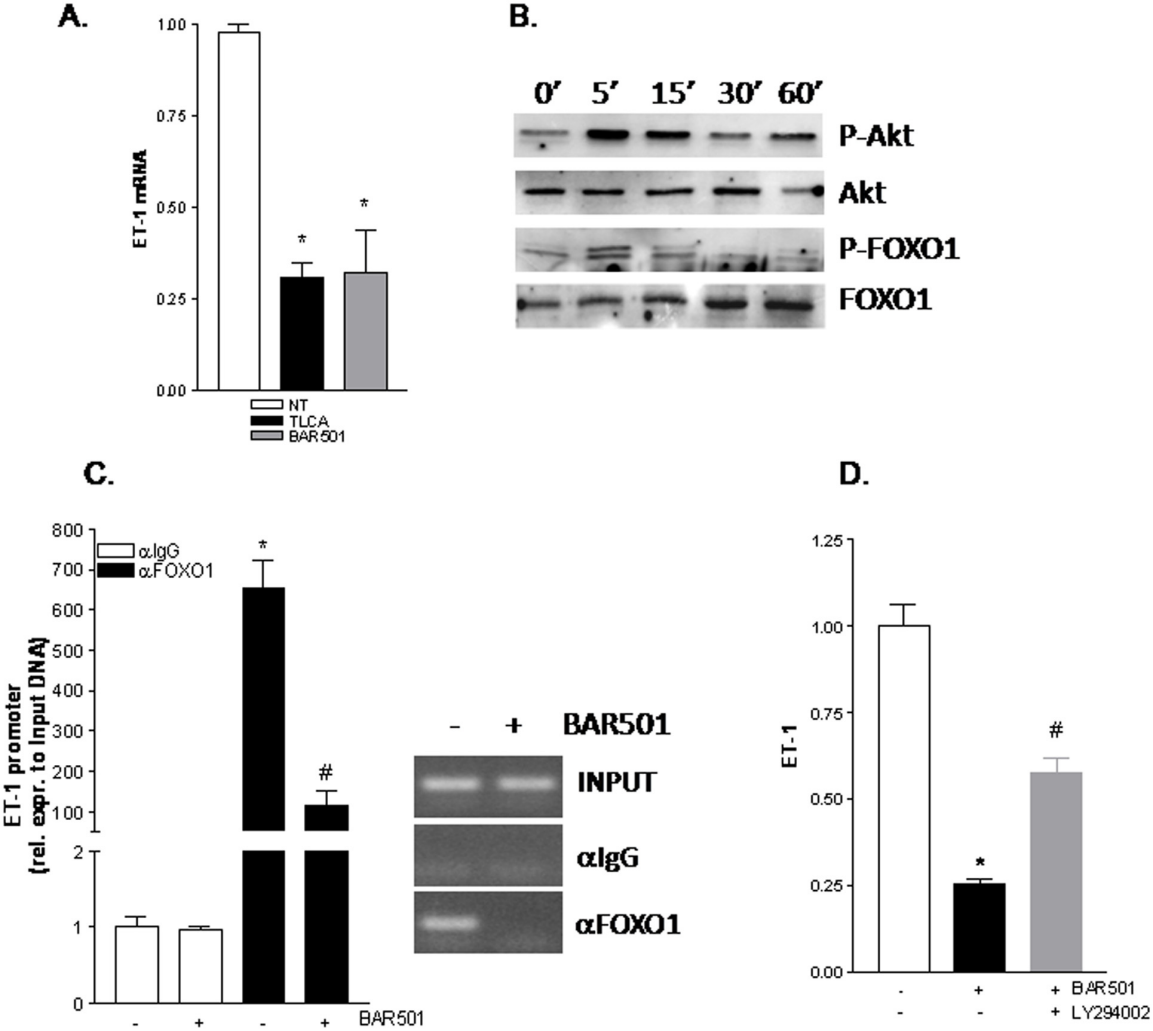
GPBAR1 activation down-regulates ET-1 expression in human LSEC by displacing FOXO1 transcriptional complex from the ET-1 promoter. (A) Serum starved LSEC were stimulated 18 h with 10 μM TLCA or BAR501. At the end of stimulation CSE mRNA expression was evaluated using Real-Time PCR method. (B) representative Western blot analysis of Akt1, phospho-Akt1, FOXO1 and phospho-FOXO1 proteins in SEC exposed to BAR501 (10 μM) for 0, 5, 15, 30, and 60 min. (C) ChIP assay carried out in SEC left untreated or primed with BAR501 as described in *materials and methods*. RT-PCR was performed with specific primers flanking the FOXO1 responsive element on human ET-1 promoter. Inset of Fig 6C. Representative qualitative PCR of ET-1 promoter immunoprecipitated with an anti-FOXO1 antibody on SEC left untreated or primed with BAR501 (D) Effect of PI3K inhibitor LY-294,002 (LY) on ET-1 mRNA expression in SEC coadministered with BAR501. *p< 0.05 versus not treated (NT) cells; #*p<*0.05 versus BAR501 treated cells.

We have next investigated the molecular mechanism involved in downregulation of ET-1 by BAR501. For this purpose serum starved LSEC were exposed to TLCA or BAR501, both 10 μM. As shown in Fig 6A, RT-PCR of ET1 mRNA revealed that both TLCA and BAR501 downregulated the expression of this gene in LSEC (Fig 6A, *p<0.05 versus not treated cells). The ET-1 promoter contains a consensus sequence for FoxO1, a transcription factor that resides constitutively into the nucleus [24,25]. FoxO1 proteins are phosphorylated by Akt/PKB through the PI3K-dependent pathway, resulting in FoxO1 nuclear export and inhibition of target gene expression [26,27]. Thus, we have inspected whether BAR501 activates Akt to regulate FoxO1 expression. For this purpose serum starved LSECs were treated for 5, 15, 30 and 60 min with 10 μM BAR501 in order to analyze by immunoblot the phosphorylation status of Akt and FoxO1 proteins. As shown in Fig 6B, the phosphorylation of both Akt and FoxO1 proteins occurred as early as after 5 min. Consistent with these observations results from ChIP confirmed that FoxO1 complexes were removed from ET-1 promoter after treatment with BAR501 (Fig 6C, *p<0.05). To further investigate the role of PI3K/Akt in mediating down-regulation of ET-1 by BAR501, LSECs were co-incubated with the LY294,002, a PI3K kinase inhibitor. Exposure to LY294002 abrogated the effect of BAR501 on ET-1 gene expression (Fig 6D, *p<0.05 versus not treated cells, #p<0.05 versus BAR501 stimulated cells).

BAR501 mediated down-regulation of ET-1 was CSE independent as demonstrated by fact that co-incubating LSEC with BAR501 and propargylglicine (PAG), a CSE inhibitor failed to revert the effect of BAR501 (S2A–S2C Fig). Moreover, BAR501 was also effective in reducing the phosphorylation of STAT1 (another transcription factor involved in the regulation of ET-1 promoter) induced by IFNγ (S3 Fig). [28]

## Discussion

**Fig 7:**
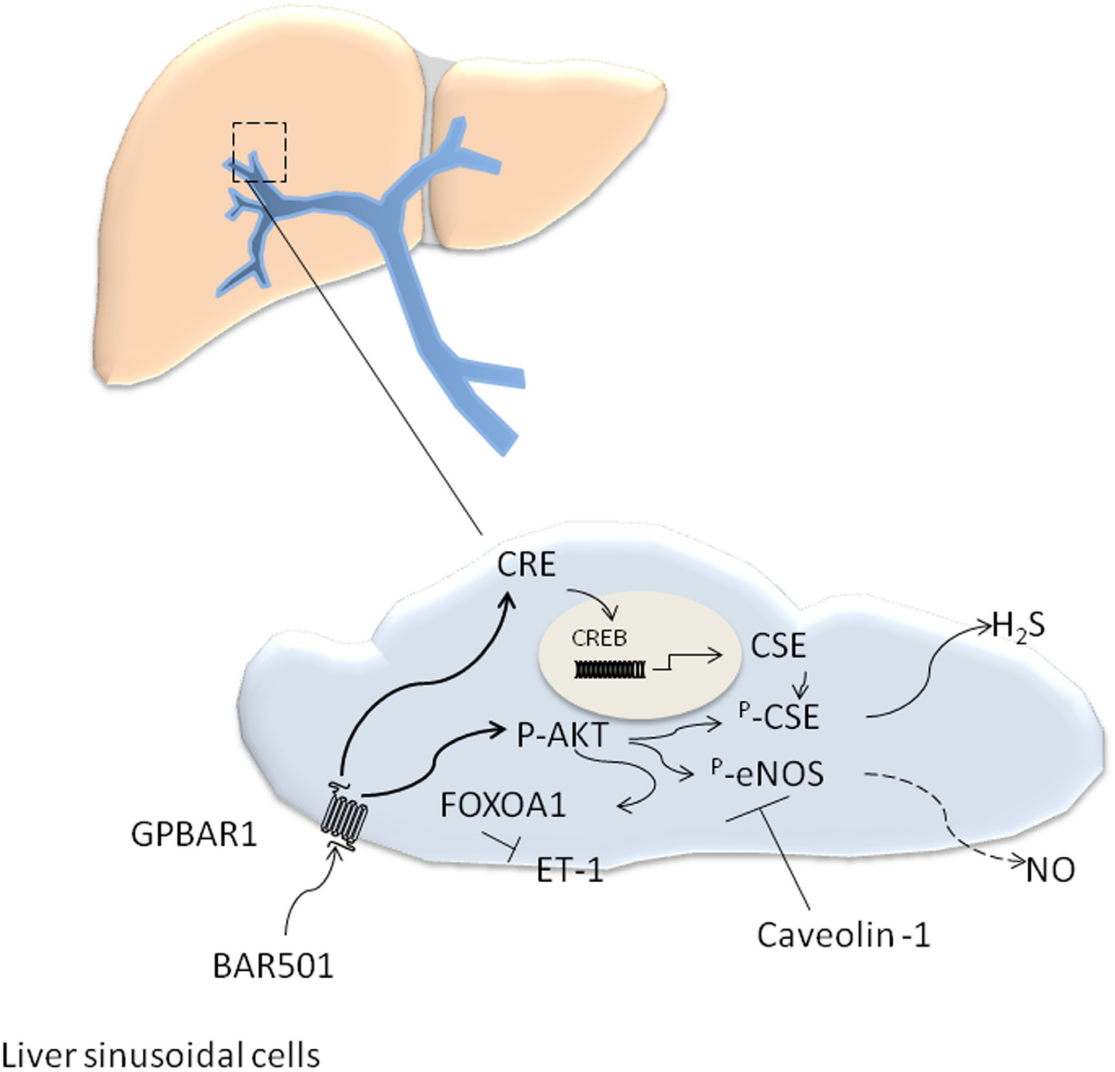
Schematic representation of the effect of BAR501 on LSEC in models of LSEC dysfunction. CCl4 and methionine feeding alter liver sinusoidal cell function. In these settings LSEC express high levels of endothelin 1 (ET-1) along with reduces eNOS activity due to enhanced binding of eNOS with caveolin-1, and reduced expression of CSE, a H2S-generating enzyme. Activation of GPBAR1 by BAR501, increases CSE expression by CRE-mediated activity, and causes both eNOS and CSE phosphorylation by AKT-mediated mechanism. In addition, AKT-driven phosphorylation of FOXOA1, attenuates ET-1 production. However since eNOS is bound to caveolin 1 the generation of nitrite/nitrate do not increase. Thus, activation of GPBAR1 in CCl4-treated mice, leads to eNOS-independent reduction of intravascular resistance, that is mostly mediated by inhibition of ET-1 and increased release of H_2_S.

GPBAR1 is a G protein coupled receptor for secondary bile acids highly expressed in entero-hepatic tissues, spleen and immune cells [1]. In the liver, expression of GPBAR1 is restricted to non parenchymal cells, specifically LSEC and Kuppfer cells [1,3]. In LSEC activation of GPBAR1 by LCA causes eNOS phosphorylation, a mechanism that could be of functional relevance in regulating intrahepatic vasomotor activity [29]. A conundrum for exploitation of this mechanism in the setting of portal hypertension, however, resides in the fact that in liver cirrhosis an enhanced binding of eNOS to caveolin-1 [6–9,30,31], alters the post-translational handling of the protein in LSEC leading to impaired generation of NO (Fig 7), a mechanism that is deemed essential for development of portal hypertension in patients with liver cirrhosis [6–9,30–33].

GPBAR1 is also expressed in the systemic vascular system and its activation mediates the vasodilatory effects of secondary bile acids [1, 10]. We have recently shown that vasodilation caused by LCA in large conductance vessels (aortic rings) requires an intact expression of this receptor [10]. Additionally, activation of GPBAR1 in arterial and venular endothelial cells regulates the expression/activity of CSE an enzyme that is critical for the generation of H_2_S [10]. Consistent with this finding, CSE inhibition by PAG reverses GPBAR1 agonism blunts the vasodilatory effect of LCA in wild type mice, but not in Gpbar1^-/-^ mice [10]. The functional relevance of this pathway is further highlighted by the observation that L-NIO, a non-selective inhibitor of eNOS and iNOS, had no detectable effect on aortic vasodilation caused by GPBAR1 ligation in mice [10].

Here, we report the pharmacological characterization of BAR501, a UDCA derivative endowed with a potent agonistic activity on GPBAR1 (Fig 7). Using the model of isolated and perfused rat liver we demonstrate that BAR501, effectively counteracts vasoconstriction caused by NE and methoxamine in intact rats, and that this effect relays on H_2_S since it was completely reversed by treating rats with PAG, a CSE inhibitor. Further on, BAR501 protects against development of portal hypertension in rodent models of liver injury and endothelial dysfunction. The portal hypertension that develops in rodents exposed to CCL_4_ is the results of extracellular matrix deposition and endothelial dysfunction due to two major mechanisms, i.e. insufficient NO production and enhanced ET-1 generation [4,6–9,15–18,30–33]. We have previously shown that activation of FXR, by 6-ECDCA (INT-747/obeticholic acid) protects against development of portal hypertension in rodent models of liver cirrhosis by reducing liver fibrosis and enhancing, among other mechanisms, the generation of H_2_S [18, 34–36]. In the present study we report that activation of GPBAR1 in mice administered CCL_4_, failed to attenuate liver fibrosis, but protected against development of portal hypertension. The fact that BAR501 failed to reduce liver collagen deposition, was expected, since GPBAR1 is not expressed by hepatic stellate cells [3]. At molecular level, we found that, while treating CCl_4_ mice with BAR501, had no effect on TGFβ1, collagen α1 and αSMA (i.e. on tissue markers of liver fibrosis), the GPBAR1 agonist effectively increased the expression of CSE without modulating the expression of eNOS and iNOS genes. Consistently, BAR501 increased the liver activity of CSE but had no effect on liver nitrites/nitrates. The lack of effect of BAR501 on eNOS, is likely due to the elevation of caveolin-1, we have observed in this model [6–9, 30–33].

Because data from the CCl_4_ model suggest a putative role of GPBAR1 in regulating liver microcirculation at the level of LSEC, we have further examined whether activation of GPBAR1 protects against development of portal hypertension in a model of liver endothelial injury, caused by feeding mice with methionine. This model leads to endothelial dysfunction, that is mostly linked to defective generation of NO [15–17]. We have previously shown that H_2_S donors protect against endothelial dysfunction caused by methionine in a NO-independent manner [16–18]. Results from current investigations demonstrate that BAR501 effectively attenuates development of increased vascular tone in methionine feed mice, but this protection results from both GPBAR1-dependent and GPBAR1-independent mechanisms. Indeed, BAR501 attenuates changes in portal pressure in an equal extent in both Gpbar1^+/+^ and Gbar1^-/-^, thought that different molecular mechanisms were involved. Indeed, in Gpbar1^+/+^ mice feed methionine, administration of BAR501, increased the expression of CSE, eNOS and iNOS, while these effects were not observed in mice harboring a disrupted Gpbar1. These results, are fully consistent with the view that activation of GPBAR1 effectively regulates H_2_S and NO production by LSEC. In contrast to the CCl_4_ model, however, endothelial dysfunction caused by methionine feeding, is not due to an enhanced production of ET-1 or caveolin-1.

The fact that BAR501, reduces portal pressure in Gpbar1^-/-^ administered methionine could be linked to the UDCA scaffold. Indeed, there is evidence that UDCA might be effective in reducing portal pressure is experimental models of liver injury, such as the bile duct ligated model [37].

The molecular mechanisms involved in the portal pressure-lowering activity of BAR501, was examined *in vitro* using LSEC. Using human primary LSEC we found that exposure to BAR501, along with other GPBAR1 ligands, increases the expression of CSE and that this effect results from both genomic and non-genomic effects. Previous studies have shown that CREB, a cyclic AMP response element (CRE)-binding protein is activated following GBBAR1 ligation by bile acids [1]. We have previously demonstrated that the CSE promoter contains two CRE elements that are conserved across the species [10]. Here, we have determined that GPBAR1 activation by BAR501 increases the transcriptional activity of human CSE promoter, while ChIP experiments provided robust evidence that BAR501 recruits CREB in its active form to a region of human CSE promoter that contains the two CREB binding sites, resulting in a functional increase of CSE activity.

In addition, data shown in Fig 5F and 5G, provide evidence that BAR501, exerts non genomic effects in LSEC, causing an Akt-dependent phosphorylation of CSE and eNOS. Taken together these results provide a molecular explanation to the potent vasodilatory activity exerted by BAR501 in the liver microcirculation. Importantly, while BAR501, causes eNOS phosphorylation, this effect does not explain the vasodilatory effects observed in the CCl_4_ model, because in this model the eNOS is sequestered by caveolin-1 (Fig 7).

One important observation made in this study, was the demonstration that exposure to a GPBAR1 agonist, negatively regulates ET-1 expression in cirrhotic livers. Because regulation of ET-1 has a major clinical readout [4], we have examined whether BAR501 directly regulates ET-1 expression in LSEC. The results of these experiments demonstrate that GPBAR1 agonism results in a Akt-dependent phosphorylation of FoxO1. Previous studies have shown that FoxO1 functions as a coactivator for ET-1 gene transcription and is constitutively recruited to the ET-1 promoter [24,25]. In contrast, FoxO1 phosphorylation disrupts the co-activator complex [26]. Data shown in Fig 6 demonstrate that BAR501, phosphorylates FoxO1 in a Akt-dependent manner and causes its release from the ET-1 promoter, thus blocking ET-1 transcription.

In conclusion, we have demonstrated that BAR501, a UDCA derivative, endowed with robust agonistic activity on GPBAR1, exerts portal pressure-lowering effects in rodent models of portal hypertension by directly regulating the expression/activity of CSE and eNOS in LSEC. BAR501 might represent a novel approach to attenuate hemodynamic changes in patients with liver cirrhosis.

## Supporting Information

S1 Fig Administration of BAR501 (30 or 40 mg/Kg) failed to reverse fibrosis in CCl_4_ administered mice. C57BL6 mice were treated for 3 weeks with CCl_4_ or with the combination of CCl_4_ plus BAR501 (30 and 40 mg/Kg body weight). Effect of BAR501 on (A) portal pressure, (B) AST, (C) ALT, (E) COL1α1 mRNA, (F) αSMA and (F) TGFβ1. Results are the mean ± SE of 4–8 mice per group. *p<0.05 versus wild type mice. #p<0.05 versus mice treated with CCl_4_.
(TIF)

S2 Fig CSE inhibitor propargylglicine (PAG) did not affect BAR501 mediated down-regulation of ET-1. Serum starved LSEC were exposed to BAR501 or to the combination of BAR501 and PAG for 18 h. Relative mRNA expression of ET-1 (A) and CSE (B) was assayed by Real-Time PCR. (C) Effect of PAG on CSE activity. *p<0.05 versus not treated (NT) cells. #p<0.05 versus BAR501 plus PAG.
(TIF)

S3 Fig BAR501 reduces IFNγ mediated phosphorylation of STAT1. Representative Western blot analysis of STAT1 and phospho-STAT1 proteins in LSEC exposed to BAR501 (10 μM) for 18 h and treated with IFNγ (100 ng/ml) for 0, 5, 15, and 45 min.
(TIF)

S4 Fig Original RT-PCR used in the preparation of Fig 6C. (JPG)

S5 Fig Original Western blot used in the preparation of Fig 5G. (JPG)

S6 Fig Original Western blot used in the preparation of Fig 5. (JPG)

S7 Fig Original Western blot used in the preparation of Fig 5B. (JPG)

S8 Fig Original Western blot used in the preparation of Fig 5F. (JPG)

S9 Fig Original Western blot used in the preparation of Fig 6B. (JPG)

S1 Table Original raw data used in preparation of Fig 1. (XLSX)

S2 Table Original raw data used in preparation of Fig 2. (XLSX)

S3 Table Original raw data used in preparation of Fig 3. (XLSX)

S4 Table Original raw data used in preparation of Fig 4. (XLSX)

S5 Table Original raw data used in preparation of Fig 5. (XLSX)

S6 Table Original raw data used in preparation of Fig 6. (XLSX)
