# Supplementary figures and images for: Reversal of Endothelial Dysfunction by GPBAR1 Agonism in Portal Hypertension Involves a AKT/FOXOA1 Dependent Regulation of H2S Generation and Endothelin-1
Source: PLoS One. 2015 Nov 5;10(11):e0141082. doi: 10.1371/journal.pone.0141082 (PMC4634759; doi:10.1371/journal.pone.0141082)

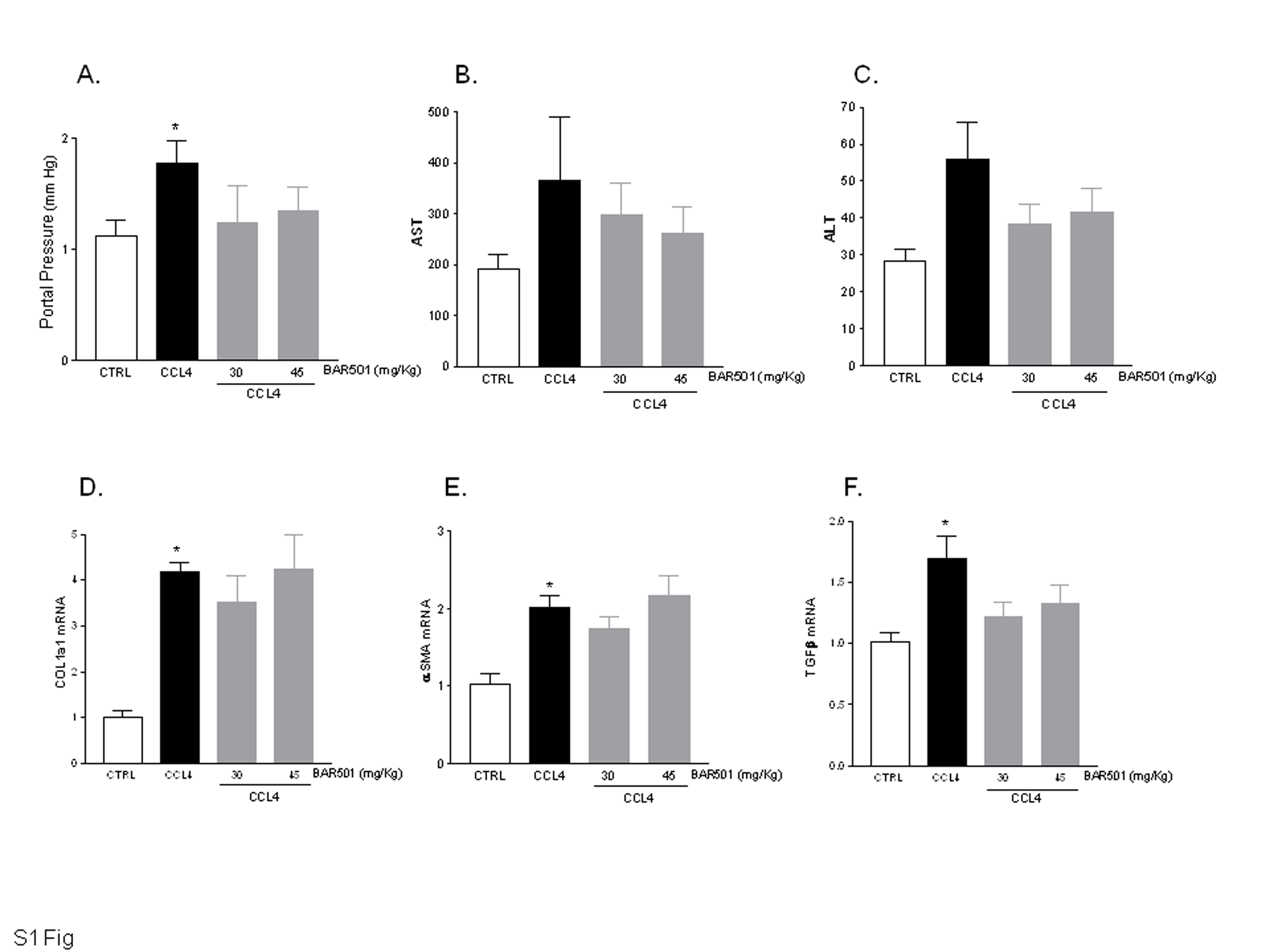

Supplement: S1 Fig — C57BL6 mice were treated for 3 weeks with CCl4 or with the combination of CCl4 plus BAR501 (30 and 40 mg/Kg body weight). Effect of BAR501 on (A) portal pressure, (B) AST, (C) ALT, (E) COL1α1 mRNA, (F) αSMA and (F) TGFβ1. Results are the mean ± SE of 4–8 mice per group. *p<0.05 versus wild type mice. #p<0.05 versus mice treated with CCl4. (TIF) [file pone.0141082.s001.tif]

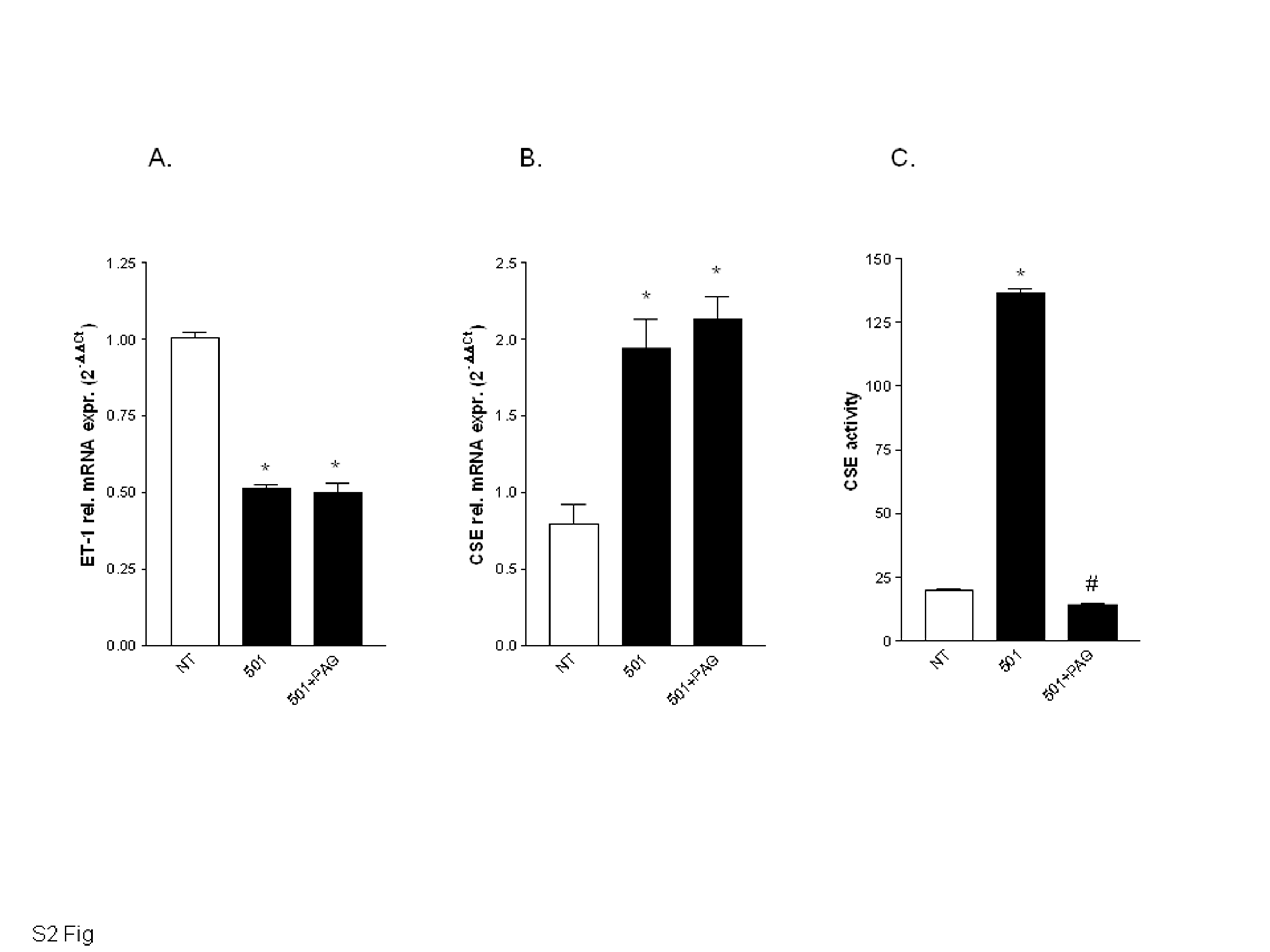

Supplement: S2 Fig — Serum starved LSEC were exposed to BAR501 or to the combination of BAR501 and PAG for 18 h. Relative mRNA expression of ET-1 (A) and CSE (B) was assayed by Real-Time PCR. (C) Effect of PAG on CSE activity. *p<0.05 versus not treated (NT) cells. #p<0.05 versus BAR501 plus PAG. (TIF) [file pone.0141082.s002.tif]

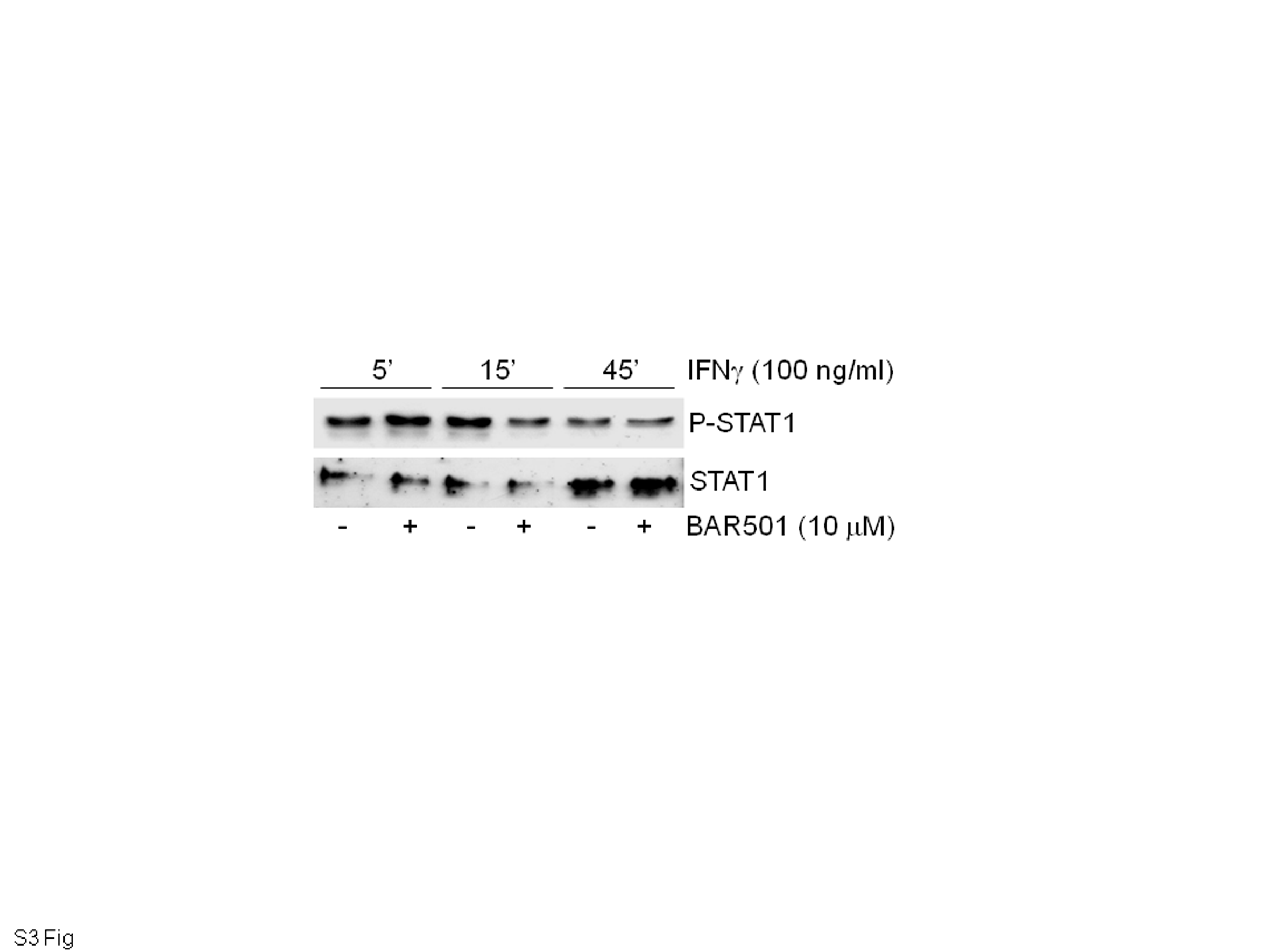

Supplement: S3 Fig — Representative Western blot analysis of STAT1 and phospho-STAT1 proteins in LSEC exposed to BAR501 (10 μM) for 18 h and treated with IFNγ (100 ng/ml) for 0, 5, 15, and 45 min. (TIF) [file pone.0141082.s003.tif]

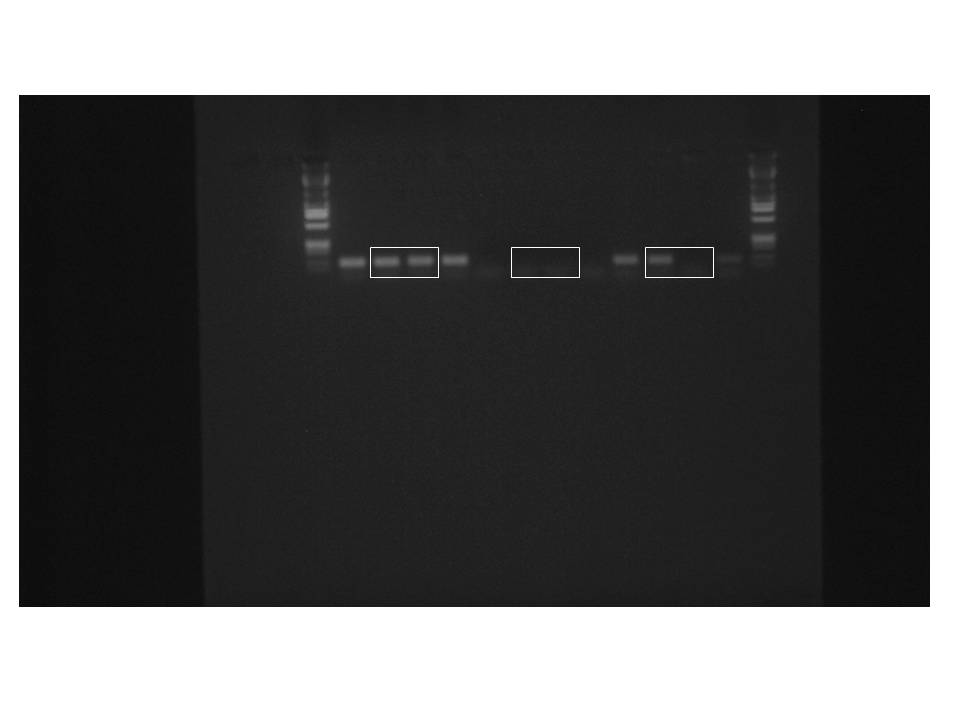

Supplement: S4 Fig — (JPG) [file pone.0141082.s004.jpg]

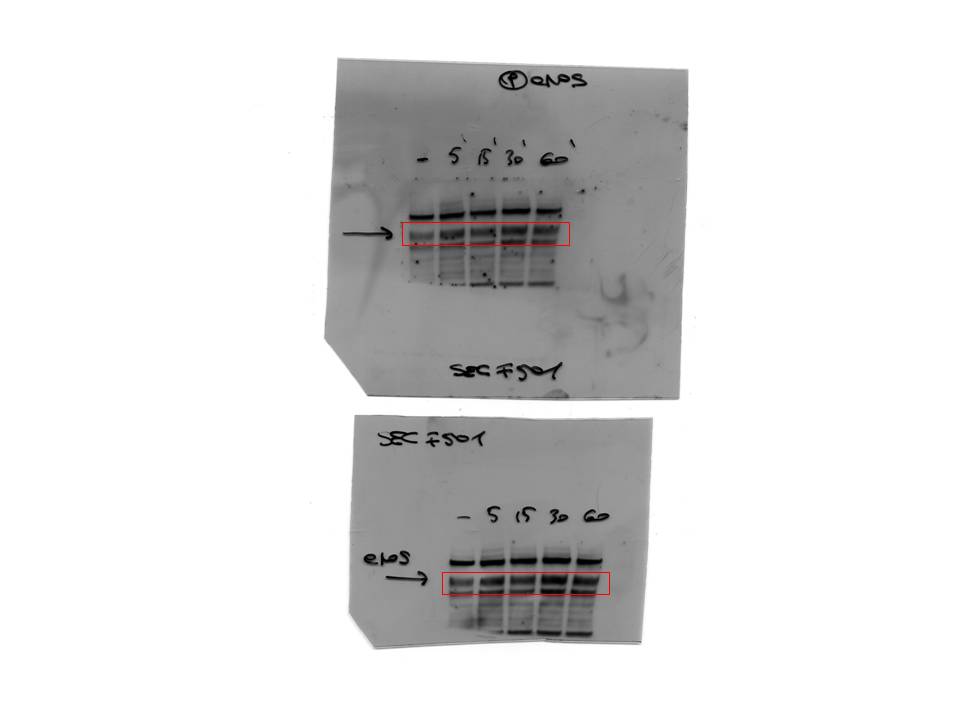

Supplement: S5 Fig — (JPG) [file pone.0141082.s005.jpg]

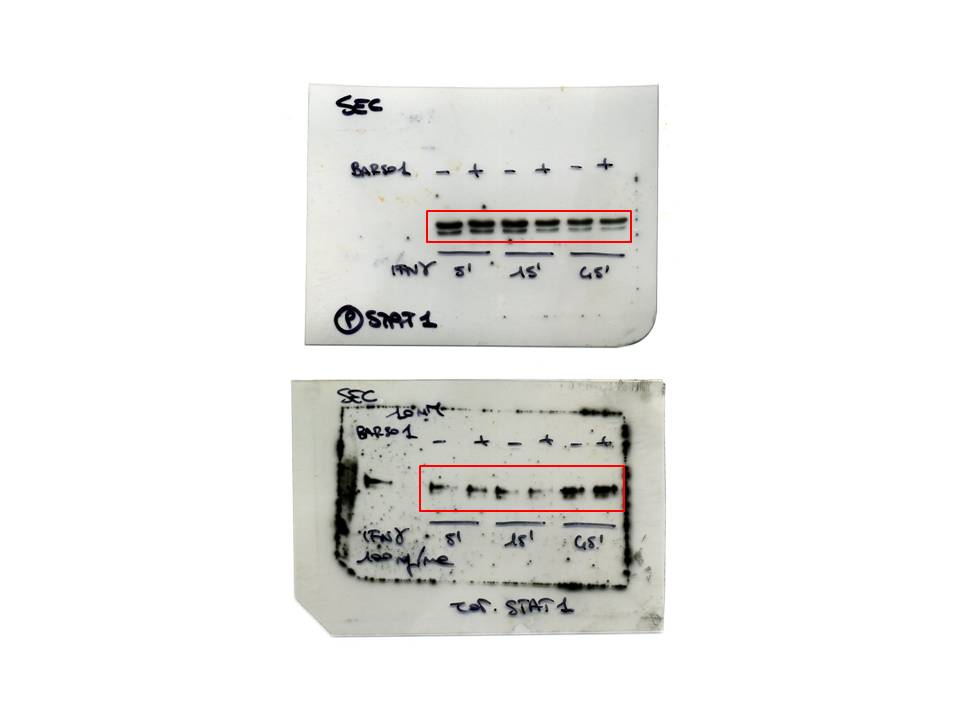

Supplement: S6 Fig — (JPG) [file pone.0141082.s006.jpg]

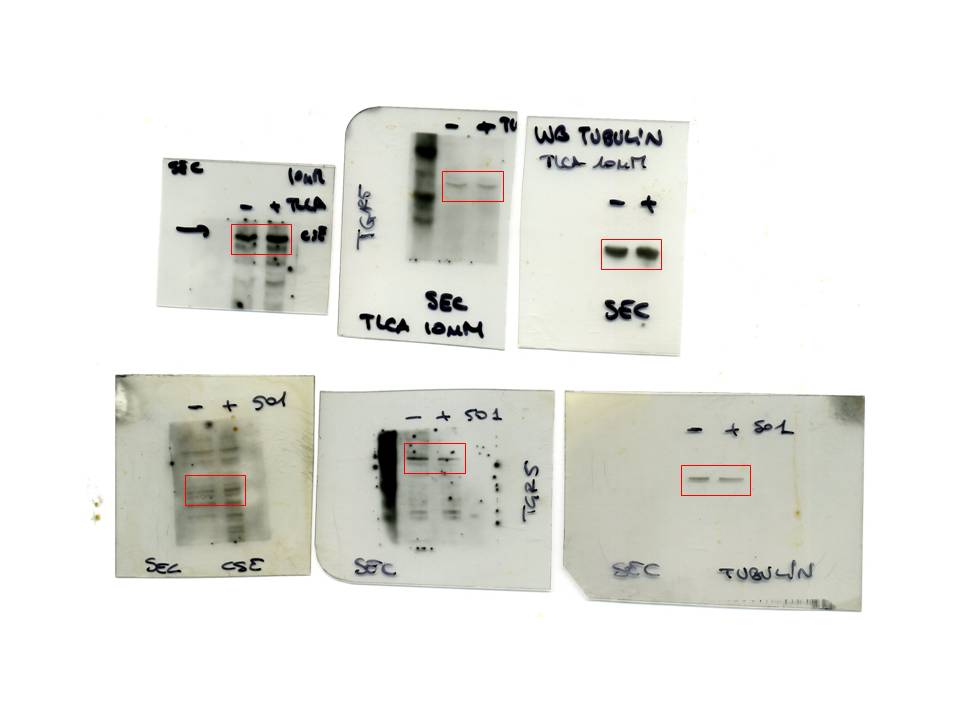

Supplement: S7 Fig — (JPG) [file pone.0141082.s007.jpg]

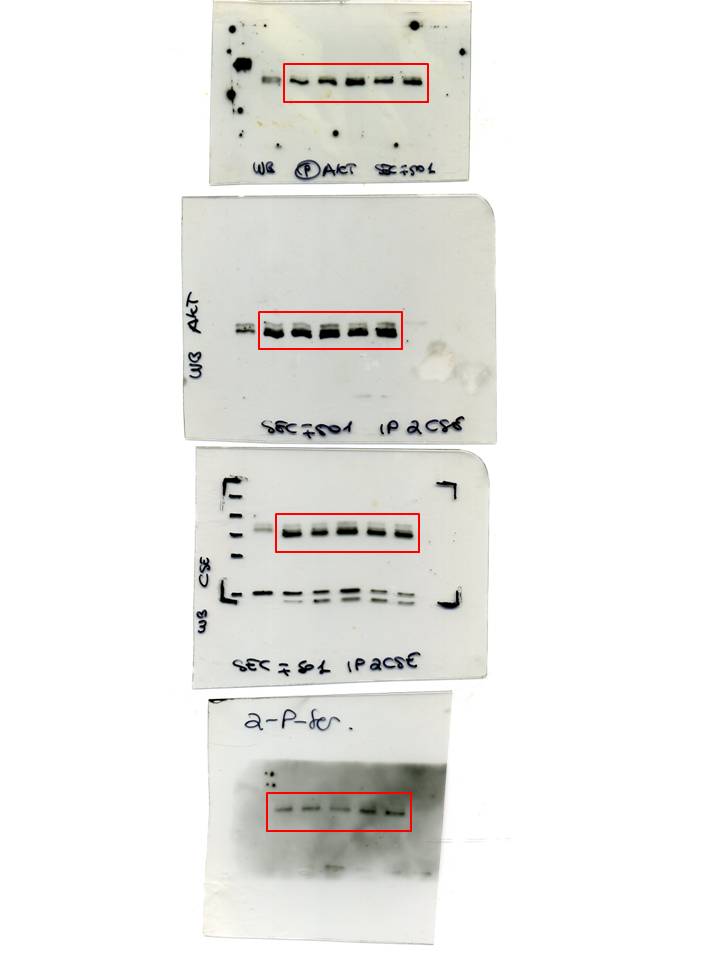

Supplement: S8 Fig — (JPG) [file pone.0141082.s008.JPG]

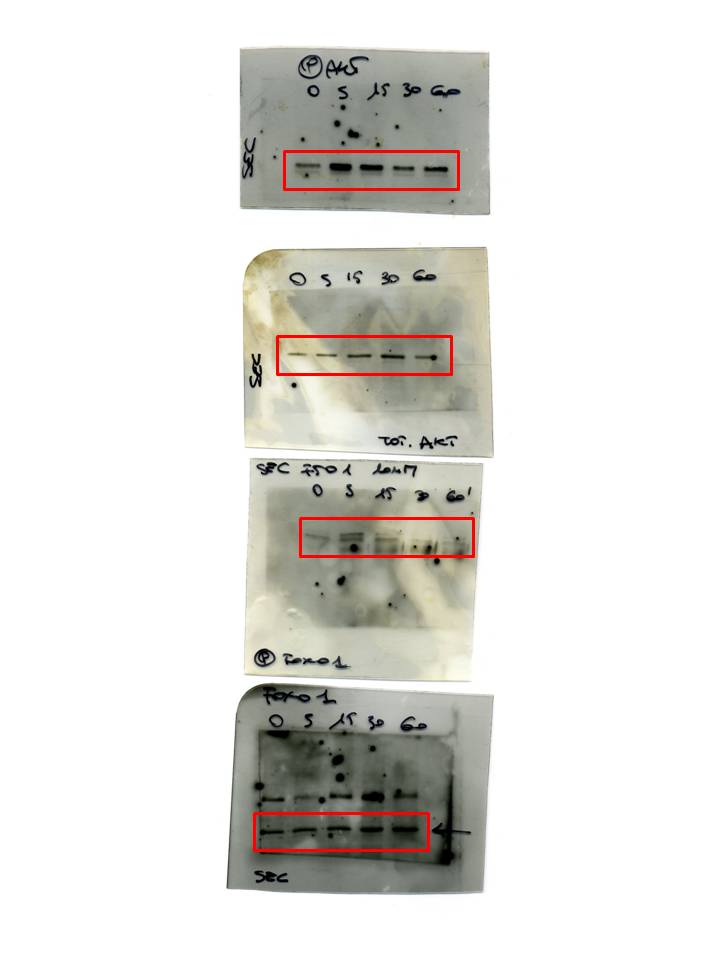

Supplement: S9 Fig — (JPG) [file pone.0141082.s009.JPG]
